# Elastin-like polypeptides as a promising family of genetically-engineered protein based polymers

**DOI:** 10.1007/s11274-014-1649-5

**Published:** 2014-04-04

**Authors:** Tomasz Kowalczyk, Katarzyna Hnatuszko-Konka, Aneta Gerszberg, Andrzej K. Kononowicz

**Affiliations:** Department of Genetics and Plant Molecular Biology and Biotechnology, The University of Lodz, Banacha Street 12/16, 90-237 Lodz, Poland

**Keywords:** Elastin-like polypeptides, Inverse transition cycling, Transition temperature, Fusion protein

## Abstract

Elastin-like polypeptides (ELP) are artificial, genetically encodable biopolymers, belonging to elastomeric proteins, which are widespread in a wide range of living organisms. They are composed of a repeating pentapeptide sequence Val–Pro–Gly–Xaa–Gly, where the guest residue (Xaa) can be any naturally occurring amino acid except proline. These polymers undergo reversible phase transition that can be triggered by various environmental stimuli, such as temperature, pH or ionic strength. This behavior depends greatly on the molecular weight, concentration of ELP in the solution and composition of the amino acids constituting ELPs. At a temperature below the inverse transition temperature (T_t_), ELPs are soluble, but insoluble when the temperature exceeds T_t_. Furthermore, this feature is retained even when ELP is fused to the protein of interest. These unique properties make ELP very useful for a wide variety of biomedical applications (e.g. protein purification, drug delivery etc.) and it can be expected that smart biopolymers will play a significant role in the development of most new materials and technologies. Here we present the structure and properties of thermally responsive elastin-like polypeptides with a particular emphasis on biomedical and biotechnological application.

## Introduction

At present, owing to the improvement in our understanding of the structure of proteins and availability of advanced tools in molecular biology, genetic and protein engineering, synthesizing DNA fragments encoding any amino acid sequences of proteins is possible with practically no restrictions (Tchoudakova et al. 2009; Chen et al. 2010). The increasing availability of various expression platforms (Conley et al. 2009; Börnke and Broer 2010; Kolotilin et al. 2012; Schipperus et al. 2012) paves the way for producing biopolymers with the desired physical, chemical and biological properties on an industrial scale. Elastomeric polypeptides are a class of protein-based polymers gaining in popularity, due to the growing demand for molecules with strictly specified properties. Encoding the polymer sequence at the gene level allows control over the composition of polymer subunits that is hard to achieve through chemical polymerization. Molecules synthesized in this way may show many properties important for their use, such as biocompatibility, biodegradability or reacting to changes in the ambient conditions in a certain way. This article is focused on elastin-like polypeptides (ELP), which are artificial biopolymers with very interesting stimuli responsive properties. Inverse temperature transition (ITT) makes them part and parcel of the rapidly developing new technologies in the field of tissue (Amruthwar and Janorkar 2013; Machado et al. 2013), protein, and material engineering or the techniques for the purification of recombinant proteins (Kwon and Cho 2012; Duvenage et al. 2013). Here we discuss current application of ELP in therapeutic peptide and drug delivery, tissue engineering, recombinant protein purification or removal of heavy metals.

## Genetically-engineered protein-based polymers (GEPBP)

One of the protein based polymers (PBP) groups currently in the focus of attention of many research teams are genetically-engineered protein-based polymers (GEPBP). These polymers are built from repeated sequences of the basic building units (blocks containing from two to several hundred amino acids) that can occur in a polymer molecule in quantities ranging from a few to a few hundred repeats (Table 1). An important advantage of the strategy for synthesizing them by use of very precisely regulated, natural molecular machinery is the possibility of introducing post-translational modifications, which are crucial due to the biological activity of some protein groups. Currently, a growing number of “tailor-made” molecules are successfully produced using natural bioreactors. Good examples of this are biomimetic proteins (Weisman et al. 2010) and proteins capable of producing materials with new properties through self-assembly (Ma et al. 2013; Maude et al. 2013). They find use in environmental engineering (Vila et al. 2011; Chu et al. 2013) pharmaceutics (Wu et al. 2009; Bessa et al. 2010) and widely understood biomedicine (Bessa et al. 2010; Gagner et al. 2014).

**Table 1:** A presentation of GEPBP (Haider et al. 2004, modified)

Class	Description	Structure	Physicochemical properties	References
ELP	Polymers containing repeats of the elastin sequence	[VPGXG]_n_, (X = every amino acid, with the exception of proline)	Chains changing their structure in response to changes in ambient temperature	Gagner et al. (2014)
SELP	Block copolymers made of silk and elastin subunits	[(GAGAGS)_m_(GVGXP)_n_]_o_ (X = every amino acid, with the exception of proline)	Possibility of creating cross-linked hydrogels	Gustafson and Ghandehari (2010)
Poly [(AG)_3_PEG]	Polymers made of repeats of nonapeptides (AG)_3_PEG	[(AG)_m_PEG]_n_	Amorphous water-soluble crystals	Ramirez et al. (2013)
Poly (CS_5_ELP)	Elastin-like blocks separated by sequences of fibronectin	[(GEEIQIGHIPREDVDYHLYP)(GVGXP)_m_]_n_ (X = every amino acid, with the exception of proline)	Conformational changes in response to changes in ambient temperature	Panitch et al. (1999)
Poly ‘‘EAK’’	Polymer repeats of EAK amino acids	[AEAEAKAK]_n_	Amyloid-like, highly stable β-sheet fibrils	Saiani et al. 2009

The strategy of biosynthesis (GEPBP) comprises several stages (Mi 2006): (1) designing a protein with the desired properties and its expression cassette, (2) building a gene encoding a protein with the appropriate amino acid composition. Short fragments of DNA (up to 100 nucleotides) are most often synthesized chemically, then assembled into longer sequences encoding the desired polypeptides with the right mass in the process of concatenation of oligonucleotides or recursive directional ligation, amongst others (Chow et al. 2008), (3) transformation of competent cells with a recombinant vector containing a gene encoding the polymer, (4) analysis the transformed cells for the presence of the introduced construct and confirming the correctness of its structure, (5) expression of the gene in the right platform and scale, (6) protein purification from the cell lysate or culture medium.

## Elastin-like polypeptides

One of the families of protein polymers obtained through genetic engineering attracting growing attention and becoming the subject of interest of many research teams around the world is elastin-like polypeptides. ELP find more and more applications in many technologies owing to their offering the possibility of directional modification of their physicochemical and biological properties.

One of the key steps in producing polypeptides with a structure appropriate for their applications is synthesis of the genes that encode them. Most strategies enabling their synthesis are based on creating libraries containing concatamers of the fragments encoding the basic structural unit of a protein polymer (Fukushima 1998; Nagarsekar et al. 2002). The main advantage of such an approach is single-step gene synthesis, although it lacks precise control of the reaction. The statistical character of the process leads to the achieving a pool of DNA oligomers with diverse length, its control is limited to changing the conditions of the reaction. For the reasons mentioned above, the more frequently used strategy for the synthesis of genes encoding elastin-like polypeptides is recursive directional ligation (RDL).

Recursive directional ligation is a process with a number of stages. Its first step consists of introducing a synthetic oligonucleotide cassette, which encodes a DNA monomer with two different endonuclease recognition sites (RE1 and RE2), to the vector. The sites are designed to generate complementary sticky ends, with these ends created after linearization of the vector with the RE1 restrictase. The next step is cutting the recombinant vector using both or just one restriction enzyme. The products created are then used for another ligation. The recombinant vector, which contains the repeated DNA monomer sequence flanked with the sites for restriction enzymes R1 and R2, does not reconstruct the restriction site for these endonucleases between them. The next rounds of digestion and ligation with the use of mono- or polymeric gene sequences lead to the generation of an increasing number of repeats of the initial DNA monomer. In this way it is possible to obtain a matrix for the synthesis of a protein polymer of the desired length. The strategy described above puts certain restrictions on the choice of sites recognized by the restriction enzymes: (1) It is necessary to choose sequences recognized by different enzymes, so as to make selective cutting with either one or two endonucleases possible, (2) the enzymes must generate complementary sticky DNA ends, (3) one of the chosen restriction sites cannot occur in the initial vector, (4) the sequences recognized by both enzymes must encode the amino acid sequence of the polypeptide. Recursive directional ligation is a quick and easy method for generating a library of genes encoding protein polymers with diverse molecular mass. Using one vector, it is possible to double the size of the gene with every round of RDL. Such an approach is the quickest method of synthesis in the case of genes with a large number of monomer units. Recursive directional ligation rivals other strategies for assembling synthetic genes encoding proteins with a repetitive structure because: (1) a polymer with the molecular mass needed for the applications in question is produced, (2) each RDL round generates identical DNA oligomers, thus eliminating the necessity of their multiple sequencing (with the exception of a small number of multimers created in the initial stages of the reaction), (3) a library of potentially useful, smaller genes is created in the process of assembling large genes, (4) monomers or oligomers encoding different peptides or proteins can be freely joined in every repeat of the reaction, which allows the structural diversity of the polymer to be increased (McDaniel et al. 2010b).

## The chemical structure of elastin-like polypeptides

**Fig. 1:**
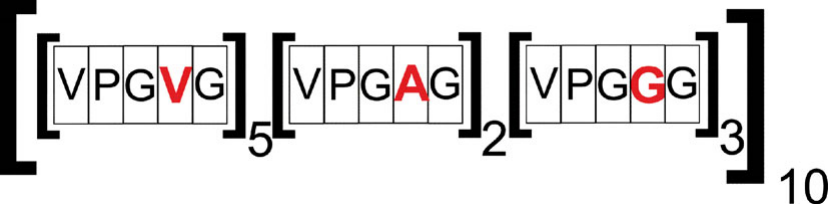
The scheme describing the structure of ELPs [V_5_A_2_G_3_-100] (Valiaev et al. 2008, modified)

Elastin-like polypeptides are polymers containing repeats of the Val–Pro–Gly–Xaa–Gly (VPGXG) pentapeptide (Fig. 1), which naturally occurs in the hydrophobic sequence of the domain of a human protein: tropoelastin, (*M*_*r*_ = 60–72 kDa) (Wise and Weiss 2009), a soluble precursor of elastin playing an important role in the process of its creation (Mithieux and Weiss 2005; Valiaev et al. 2008). Elastin is a structural protein of the extracellular matrix (created in the process of assembling tropoelastin monomers into a stable polymer structure) present in the connective tissue of all vertebrates. Its main function is to provide elasticity for large blood vessels (aorta), ligaments, lungs and skin (organs subjected to constant and reversible physiological deformations (Tsamis et al. 2013). Xaa (the so-called guest residue) in the ELP sequence can contain any amino acid, with the exception of proline (Gagner et al. 2014), proline occurring here neutralizes the characteristic and very useful properties of the polymers discussed (Trabbic-Carlson et al. 2004b). The classification of elastin-like polypeptides in the literature is based on the kind and number of amino acids occurring in the guest residue position (van Eldijk et al. 2012). In order to simplify and standardize the notation of the structure of a particular ELP, the following scheme has been agreed upon: [X_i_Y_j_Z_k_-n], where:

- X, Y, Z—single-letter symbols of amino acids occurring in the place of the guest residue in the pentamer;
- i, j, k—the ratio in which the amino acids occur in the guest residue position in the basic structural unit;
- n—the total length describing the number of polypeptides contained in the given ELP.

There are many variants of elastin-like polypeptides, containing repeats of different amino acid sequences, such as penta-: KGGVG (Martino and Tamburro 2001), VGGVG (Flamia et al. 2004, 2007), GVGVP (Swierczewska et al. 2008), hepta-: LGAGGAG or nona-peptides: LGAGGAGVL (Spezzacatena et al. 2002). Some polymers containing patterns coming from the elastin sequence, show its characteristic properties. This paper focuses only on elastin-like polypeptides containing repeats of the ELP[VPGXG-n] pentapeptide, which are most often described in the literature.

## Physical properties

**Fig. 2:**
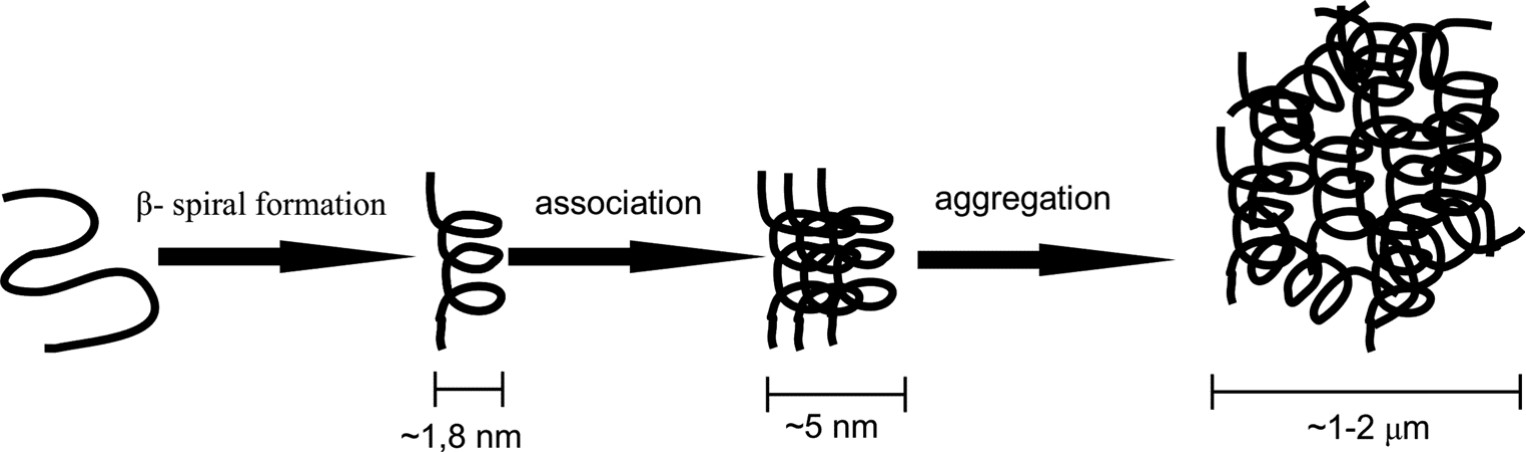
The ELP aggregation mechanism (Rodriguez-Cabello et al. 2007, modified)

Elastin-like polypeptides belong to one of the three classes of thermosensitive biopolymers (Mackay and Chilkoti 2008), whose properties are subject to change depending on the ambient temperature. Aqueous solutions of elastin-like polypeptides show a lower critical solution temperature (or LCST), which above the so-called phase transition temperature (T_t_) results in the transition of elastin-like polypeptides from a soluble to an insoluble form (van Eldijk et al. 2012) in a narrow range of temperatures (~2 °C) in a reversible process known as coacervation. The phase transition temperature is maximum value defined as the temperature in which ELP aggregation occurs, causing solution turbidance to increase to half its initial value, accompanied by an increase in the temperature of the solution (Meyer and Chilkoti 1999). In solutions with a temperature lower than T_t_, free polymer chains remain in an unordered state showing full hydration (the soluble form). In solutions with temperatures exceeding T_t_, the situation is different: polymer chains show a more ordered structure (known as the β-spiral), stabilized by hydrophobic interactions (Rodriguez-Cabello et al. 2007) and intramolecular type β structures increasing the association of polymer chains (Serrano et al. 2007). Figure 2 depicts the described process.

The process is reversible, with a decrease in temperature ELP undergoing transition to the soluble form. It is interesting that this property remains after ELP is fused to another protein. A paper by Kim et al. (2005a) describes the fusion of G and L proteins with the ELP domain. The phase transition temperature level of elastin-like polypeptides depends on a few factors: the concentration of ELP in the solution, the ionic strength of the solution and the molecular mass of the polymer. Along with an increase in these parameters, the phase transition temperature decreases. The presence of different amino acids in place of the guest residue influences the level of *T*_*t*_ of a given biopolymer. A hydrophobic group in this position lowers the phase transition temperature, while a hydrophilic group increases it (the effect depends on the molar ratio of particular residues) (McMillan et al. 1999). Different substitutions of the guest residue allow ELP to be designed with the desired *T*_*t*_. Using amino acid residues containing groups susceptible to ionization results in a polymer with a *T*_*t*_ regulated by a change in pH, while using an amino acid with a chemically reactive side chain allows ELP to be conjugated with other molecules (McMillan et al. 1999; McMillan and Conticello 2000).

## Selected applications of elastin-like polypeptides

Elastin-like polypeptides find increasingly wide use in biomedicine because: (1) the phase transition temperature can be specifically “programmed”, which enables an ELP to be designed which is suitable for the intended purpose, (2) it is possible to create ELPs with a strictly specified molecular mass, which is important in their application as drug carriers, since molecular mass is a key parameter influencing the half-life of the drug in the body, (3) Expression of ELP can occur at a level high enough to become an alternative to polymers produced through chemical synthesis, (4) the property of the reverse phase transition allows purification of these peptides without the use of costly chromatographic methods.

## Application of ELPs in medicine

One of the main problems of modern oncology is often the lack of selective carriers of antineoplastic drugs, delivering the drug only to pathologically changed tissue. In most cases, only a small portion of the drug reaches its destination, while the remaining part has a negative influence on healthy cells. This makes it necessary to develop carriers that will make chemotherapy less toxic and more effective. Current research into neoplasm therapy already uses synthetic and natural soluble polymers such as liposomes (Sofou et al. 2010; Cai et al. 2014), microspheres and nanospheres (Vasir and Labhasetwar 2005; Mukerjee and Vishwanatha 2009; Montejo et al. 2010; Rajput and Agrawal 2010) and also micelles (Prabaharan et al. 2009; Wang et al. 2014). There are reasons to believe that the application of thermosensitive polymers can lead to better therapeutic effects in comparison to traditionally employed methods. This especially applies to minimizing the side effects of the drugs used. The particular properties of an ELP allow the polymer-drug conjugate to accumulate preferentially in the vicinity of the tumor and to show lower toxicity in comparison to freely operating drugs. Thanks to the reverse phase transition property and the topical use of hyperthermia, an increase in the drug penetration of the pathological tissue is possible.

## Application of ELPs in therapeutic peptide delivery

It has been proved that disturbances in c-Myc protein expression are connected to the development of many kinds of human neoplasms. To show its oncogenic activity, it has to interact with a protein Max partner (dimerization). This makes blocking c-Myc-Max dimer formation an effective method of slowing down cancer cell division, and thus an effective method for fighting neoplasms. The results of a paper by Giorello et al. (1998) point to the possibility of blocking the transcription activity of c-Myc. Based on the paper mentioned above, Bidwell III and Raucher (2005) showed the ability of elastin-like polypeptides to deliver this peptide to neoplastic cells. A paper by Massodi et al. (2010) also points to the possibility of the effective use of ELP in conjunction with the p21 and Bac peptides. The p21 mimetic peptide is capable of inhibiting the proliferation of ovarian cancer cells combined with hyperthermia. The transition temperature of Bac-ELP1-p21 in the range from 39 to 42 °C, caused the inhibition of retinoblastoma protein phosphorylation, arresting the cell cycle and activating caspases at 42 °C.

Moreover, application of cell-penetrating peptides (CPPs) can be used to overcome the transport barriers in tumor tissue, which inhibit drug delivery of polypeptides to their appropriate intracellular molecular target. CPPs are usually short peptides that have the capacity to ubiquitously cross (alone or linked to protein) cellular membranes with very limited toxicity, in an energy-dependent and/or independent way, without the necessity of recognition by specific receptors (Bechara and Sagan 2013). So far, different CPPs have been fused to ELP chains to improve their therapeutic utility. Bidwell III and Raucher (2010) compared the efficiency of various CPPs (Pen, Tat, MTS) for intracellular delivery of ELP using fluorescently labeled CPP–ELP polypeptides for flow cytometry and confocal microscopy. It was shown that penetratin (Pen) was the most efficient. The cellular association of the polypeptide was increased 1.7, 2.6, and 14.8-fold for Tat-ELP, MTS-ELP, and Pen-ELP respectively, relative to the ELP without CPP. Additionally, the results of endocytosis inhibitors applied in flow cytometric cellular uptake assays can suggest that CPP–ELP internalization occurs via a caveolae-independent endocytic mechanism.

Thermally responsive elastin-like polypeptides can be a very promising class of protein-based biopolymers for the delivery of anticancer drugs to solid tumors via systemic or local administration. These polymers possess several advantages compared to other anticancer drug delivery vehicles that appeared to be complementary and synergistic with existing technologies: (1) composition of ELPs can be precisely encoded at the gene level, (2) ELPs have the advantages and characteristics of soluble macromolecules which accumulate in tumors due to a passive targeting provided by enhanced permeability and retention effect, (3) they are thermally responsive and may be actively targeted by application of local hyperthermia,(4) the addition of CPPs to the ELP chain enhances uptake into the tumor cells, and moreover, CPPs also mediate the escape of polymers from the tumor vasculature into the tumor cells. (5) the addition of CPPs can target ELP to the desired cellular compartment (Bidwell III and Raucher 2010), (6) peptide-based biomaterials are easily degraded by the body and possess good biocompatibility, (7) ELP half-life in the plasma can be precisely controlled.

## Application of ELPs in drug delivery

Massodi et al. (2009) reported the effect of slowing down the cell adhesion of migrating tumor cells in the case of ovarian cancer cells, reducing metastasis using the Tat protein of the HIV-1 virus in conjunction with a sequence of an elastin-like polypeptide. The efficiency of thermosensitive polypeptides in delivering the drug to pathologically changed tissue has also been proved in a paper by Meyer et al. (2001). Two thermosensitive polymers were synthesized, with their *T*_*t*_ higher than the physiological temperature of the human body. The delivery of the polymer-rhodamine conjugate to the tumor was tracked with a fluorescence microscope. The results show that increasing the temperature of the vicinity of the tumor to 42 °C caused a twice greater accumulation of the conjugate in comparison to the trial without the use of hyperthermia. A considerable increase in the cytotoxicity of the drug towards neoplastic cells in conditions of a topical increase in temperature has also been proven by Bidwell et al. (2007), using ELPs in conjunction with a reactive derivative of doxorubicin. Subsequent experiments showed the cytoplasmic distribution of the drug in cells and a thermally induced caspase activation.

An interesting aspect of the use of ELPs as drug carriers is the possibility of creating block copolymers (Chilkoti et al. 2002) with various architectures (micelles, vesicles). The different properties of particular blocks of copolymers enable the convenient encapsulation of drugs. Experiments with the use of fluorescent dyes: nile red and 8-anilinonaphthalene-1-sulfonic acid showed the possibility of encapsulating small molecules in the micellar core of diblock polymer nanoparticles at a temperature above the LCST of their hydrophobic blocks (Wright and Conticello 2002). The strategy proposed by Wu et al. (2009) is based on the production of ELP nanoparticles through electrospraying with simultaneous encapsulating of the drug inside the polymeric shell. The paper quoted above presents factors influencing the diverse morphology of the ELP aggregates that are formed. It was proved that this method allows effective encapsulation of the drug in the polypeptide shell. Creating a biodegradable scaffold that can be “loaded” with an antibiotic and then used in the treatment of local infections (Adams et al. 2009) was also attempted. Experiments on encapsulating vancomycin and cephazolin showed the expected effect of antibiotics. An additional advantage of such an approach is immune neutrality of the ELP. Amruthwar and Janorkar (2012) also showed ELP as a hydrogel scaffolds that are able to release a model protein and antibiotic. Comparison of the release profiles of doxycycline and BSA showed that the release was higher at 25 °C compared to that at 37 °C only for the higher concentration of BSA (1 % v/v). These data demonstrate that the drug’s molecular weight and loading concentration affect the release kinetics.

Elastin-like polypeptide-based dendrimers, which are synthetic macromolecules with a unique structure, can also be used for drug delivery and as a potential scaffold for peptides. Kojima and Irie (2013) have synthesized a novel type of temperature-dependent drug carrier by conjugating Ac–Val–Pro–Gly–Val–Gly to a dendrimer. They demonstrated that these elastin-mimetic dendrimers could be a potent drug carrier, because they are able to release a model drug, rose Bengal, from their structure. Moreover, their temperature-dependent properties can be controlled by peptide chain length, peptide sequence, and dendrimer generation.

Elastin-like polypeptides as a promising family of genetically-engineered protein-based polymers for the drug delivery in cancer therapy, can be designed to best exploit the relationship between the transition temperature (*T*_*t*_) of the ELP and body temperature (*T*_*b*_). In general, there are four different strategies for drug delivery: (1) hydrophilic ELPs with a *T*_*t*_ much greater than *T*_*b*_ are used as soluble, hydrophilic macromolecular carriers of conjugated drugs to take advantage of the EPR effect for systemic delivery. (2) ELPs exhibiting a *T*_*t*_ between 37 and 42 °C are systemically delivered in combination with externally applied, local hyperthermia. This strategy assumes the aggregation of drug carriers in the tumor vasculature that enhances its concentration and increases drug diffusion into the tumor upon return to normothermia. (3) ELP block copolymers are designed to form nanoparticle micelles in response to hyperthermia, resulting in a multivalent display of ligands on the micelle corona. (4) intratumoral injection of drugs utilizing ELPs for local delivery. In this approach biopolymers are designed to be soluble and injectable at room temperature but to coacervate at physiological temperatures, increasing the length of exposure of the tumor to conjugated drug (McDaniel et al. 2010a).

## Application of ELPs in tissue engineering

The properties of elastin-like polypeptides described above make them an excellent material that could be widely used in tissue engineering and regenerative medicine. Creating scaffolds made of ELPs means that tissue engineering is achieved by way of three main methods: (1) creating coacervates (Asai et al. 2012), (2) physical cross-linking (Wu et al. 2005), (3) chemical cross-linking (Lim et al. 2007). The use of elastin-like polypeptides in treating articular cartilage damage is especially interesting, due to the widespread nature of this ailment.

**Fig. 3:**
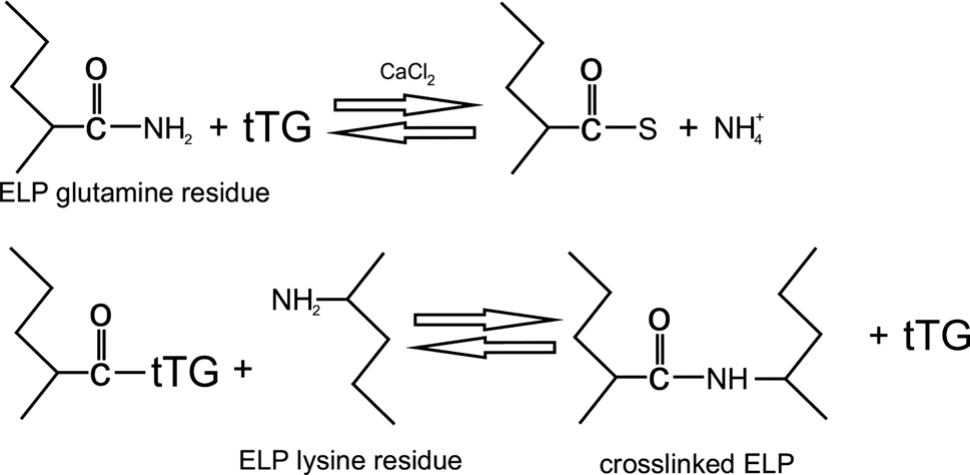
The scheme of the reaction of ELP cross-linking with the use of transglutaminase (McHale et al. 2005, modified)

Articular cartilage is a very specific tissue, as it has no blood vessels, no lymphatic drainage or innervation, and covers the subcartilaginous layer of bones that create joints. It is a living tissue, highly durable and elastic. Thanks to its very specific structure, articular cartilage has the ability to absorb the forces acting on it, at the same time minimizing friction between the moving joint surfaces. Articular cartilage is composed of cartilaginous cells known as chondrocytes and the intercellular substance—cartilaginous matrix, containing a few types of collagen, predominantly type II collagen and a few types of proteoglycans and non-collagenous proteins. Damage to this tissue is a serious problem affecting many people regardless of their age. Unfortunately, articular cartilage has negligible regenerative ability. Pathology of articular cartilage in the form of degeneration at different stages of development is the third most common illness in Central and Eastern Europe. For a long time now, attempts have been made to create an effective method for treating cartilaginous tissue loss. Despite their technical advancement, the effectiveness of most of these methods is unfortunately insignificant. Currently, the most effective method for treating different types of damage is transplanting chondrocytes using biomaterials that can be described as scaffolds. These are most often built from different types of biopolymers. Elastin-like polypeptides are among the biomaterials used in treatment of articular cartilage damage, their effectiveness was proved by McHale et al. (2005). The experiment entailed synthesizing a hydrogel made of ELP in which chondrocyte cells were encapsulated, then their ability to produce a cartilaginous matrix in vitro was assessed. The results of the experiment are promising, since chondrocytes are effectively encapsulated by ELP and, creating a sort of scaffold, they contribute to the accumulation of the extracellular matrix containing type II collagen and glycosaminoglycans. It was also proved that making a covalent modification to ELP by creating cross-links using transglutaminase (Fig. 3) increases the rigidity of the material obtained.

The earlier works by the authors mentioned above also show that some properties of the ELP hydrogel can be precisely controlled by means of setting the molecular mass, the concentration and the content of the reactive groups in polypeptides. A great advantage of using elastin-like polypeptides is the possibility of producing them with high efficiency. ELP can be produced with an efficiency of even 500 mg/l of the bacterial culture, while the efficiency of producing other biopolymers does not exceed 10–20 mg/l (McHale et al. 2005). It is very important that the purification process of elastin-like polypeptides is simple and effective.

Janorkar et al. (2008) indicated that chemically modified tissue culture polystyrene (TCPS) with chemically derivatized ELPs enables control over the in vitro morphology and liver-specific function of hepatocytes. Conjugation of ELP with polyacrylic acid and polyethyleneimine can strongly influence aggregation, morphology or differentiated function of primary rat hepatocytes. It was demonstrated that hepatocytes cultured on ELPs conjugated to positively charged polyelectrolytes form spheroids with increased production of urea and albumin, compared to cell culture on ELP or ELP conjugated with negatively charged polyelectrolyte. These data show the ability to use ELP in liver tissue regeneration.

Elastin-like polypeptides have also been applied in ocular tissue engineering. Martínez-Osorio et al. (2009) tested recombinant ELP polymers as scaffold for epithelial cells. These engineered polymers, containing two elastin-derived units, fibronectin CS5 domains enclosing the cell attachment sequence, and a proteolytic domain were absorbed to glass surfaces to promote epithelial cell attachment, proliferation, and retention of the differentiated phenotype. These positive preliminary data will allow the development of polymer-based scaffolds to be used in ocular surface tissue engineering.

Elastin-like polypeptides can also be a part of the technology of vascular graft tissue engineering. Nicol et al. (1992) polymerized X20-poly-(GVGVP), the gamma-irradiation cross-linked elastomeric matrix based on pentamer Val–Pro–Gly–Val–Gly, and X20-poly[n(GVGVP), (GRGDSP)] containing the covalently incorporated cell adhesion sequence Arg–Gly–Asp–Ser (RGDS) to support the adhesion and growth of bovine aortic endothelial cells and of bovine ligamentum nuchae fibroblasts. These authors demonstrated that ELP polymers without cell recognition sequences are poor substrates for cell adhesion and bioactive ELPs are required for their application to vascular graft tissue engineering.

Liu and Tirrell (2008) described a simple method for making cross-linked aECM films that are suitable for cell studies. In this work the adhesion, spreading, and migration of human umbilical vein endothelial cells on crosslinked films of artificial extracellular matrix were examined. They examined an artificial extracellular matrix composed of elastin-like structural repeats and fibronectin cell-binding domains (ECM-RGD) and covalently attached poly(ethylene glycol) to aECM (ECM-RDG-PEG). This work demonstrated that PEGylated protein substrates show low levels of nonspecific cell adhesion but retain the ability to bind cells in a sequence-specific manner. Moreover, depending on the concentration of authentic cell-binding domains in the scaffold, it is possible to modulate cell adhesion and spreading. This biomaterial designed for use in small-diameter vascular tissue can find application and utilization in soft-tissue engineering, especially in small-diameter vascular grafts.

## For protein purification

**Fig. 4:**
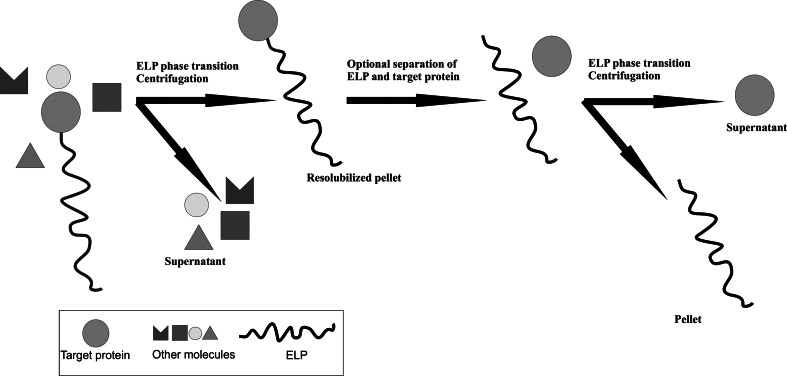
The scheme of protein purification in inverse transition cycling (ITC) (Urry and Pattanaik 1997, modified)

The rapidly increasing number of recombinant proteins produced necessitates intensive research on simple, cheap and efficient ways of purifying proteins on the laboratory and industrial scale. One widely used method entails creating hybrid proteins, in which one of the protein partners plays the role of a tag (Zhao et al. 2013) The use of elastin-like polypeptides seems to be an alternative to the traditional methods of protein purification. Comparison of ELPs and other affinity tags is shown in Table 2. ELPs are used, among others, in a non-chromatographic method known as inverse transition cycling or ITC. The process is simple and its course is illustrated in (Fig. 4).

**Table 2:** Comparison of ELPs tag and other affinity tags

Tag	Size (aa)	Size (kDa)	Sequence	Properties	References
His-tag	6	0.84	HHHHHH	Binding to immobilized metals under denaturing or native conditions	Knecht et al. (2009)
FLAG	8	1.01	DYKDDDDK	Binding to several specific anti-FLAG monoclonal antibodies	Futatsumori-Sugai et al. (2009)
Streptag II	8	1.06	WSHPQFEK	High specificity and affinity towards the protein streptavidin	Ayala et al. (2013)
ELP	Different size	Different size	[VPGXaaG]_n_	Undergo an inverse temperature phase transition	Kwon and Cho (2012)
Chitin binding domain	51	5.59	TNPGVSAWQVNTAYTAGQLVTYNGKTYKCLQPHTSLAGWEPSNVPALWQLQ	Affinity purification of the fusion protein on a chitin resin	Guan et al. (2013)
Maltose binding protein	396	40.00	Protein	Amylose affnity purifcation	Miklos et al. (2013)

The ITC process depends on increasing the ionic strength and/or temperature of the solution containing the cell lysate in order for ELP aggregation with the protein fusion partner to occur. The following process of sample centrifugation allows the ELP-protein fusion to be separated from the impurities. All of the denatured and aggregated biomolecules constituting the impurities can be separated from the ELP-protein fusion in another centrifugation. The whole ITC process can be repeated until the expected purity of the preparation is reached. Comparison of traditional (chromatographic) protein purification methods with the ITC using ELPs was conducted by Trabbic-Carlson et al. (2004a). The authors evaluated the effectiveness of the protein purification method involving inverse transition cycling with the immobilized metal affinity chromatography. The results clearly demonstrate that the protein purification method based on polypeptides has an advantage over the traditional chromatographic process because: (1) ITC is a non-chromatographic method, and no special equipment is required to perform it, (2) ITC is easy to scale-up and the process is highly efficient, (3) ITC is quick and easy to implement.

It was also found that the ELP fused to proteins stabilizes the fusion partner, preventing aggregation and denaturation in high concentrations. Trabbic-Carlson et al. proved that the purification of chloramphenicol acetyltransferase (CAT), blue fluorescent protein (BFP), thioredoxin (Trx) and calmodulin (CalM) by ITC can reach the same level of purity as purification by IMAC with an additional increase in efficiency. There is also the possibility of applying indirect ITC by creating a fusion ELP-protein with an affinity to the molecules that we want to purify (Kim et al. 2005b). This approach eliminates the need to remove the ELP tag in the final step of the whole process. ITC seems to be a good alternative to traditional methods of protein purification, suggesting that this technique will be used routinely, especially as it allows the recovery of proteins present even at low concentrations (Christensen et al. 2007). The applicability of this method in combination with self-excision intein domains makes the ITC-based process more efficient (Banki and Wood 2005; Banki et al. 2005).

## Removal of heavy metals

**Fig. 5:**
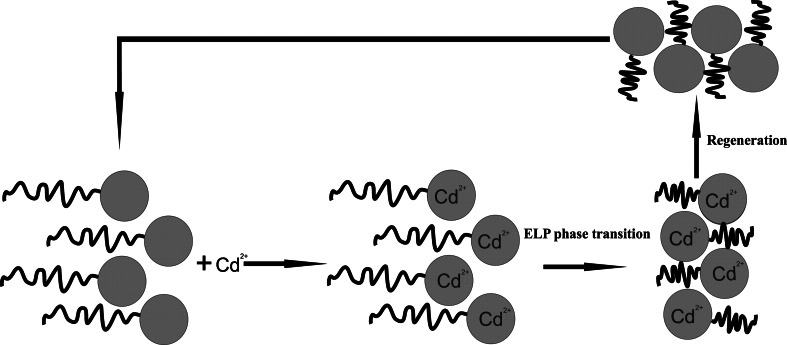
The schematic representation of heavy metal removal process with application of ELP (Kostal et al. 2005, modified)

In recent times we have seen the appreciation of research focused on reducing environmental pollution and the remediation of contaminated areas. A research team from the Department of Chemical and Environmental Engineering, University of California has shown the application of elastin-like polypeptides for removing heavy metals from a contaminated environment. This method, called “Polymer Filtration”, utilizes water soluble polymers for the chelation of heavy metals and removing them from the polluted area. This technology is very effective, and what is more, it can be used on a large scale. An additional advantage of the method is the possibility to regenerate polymers in order to use them in subsequent cycle (Fig. 5).

Kostal et al. (2005) conducted their studies on the application of ELP in binding heavy metals. The fusion of MerR protein (bacterial protein having a high affinity for mercury ions) with the ELP domain were obtained, which allows water polluted with mercury to be purified to a level acceptable for consumption. The process of purifying contaminated water or soil is relatively simple and it can be expected that its optimization makes it possible to use it routinely. Lao et al. (2007) also utilized ELPs to purify soil polluted with cadmium. These authors designed a fusion protein composed of ELP and synthetic phytochelatin, thus achieving selective removal of metals from the contaminated soil. The existence of proteins that bind a wide variety of heavy metals with high specificity (such as metallothionein and phytochelatins) makes this technology very useful in the removal of a wide range of xenobiotics from natural ecosystems.

The specific and extremely useful properties of ELPs make them a component of many new and more sophisticated technologies. New reports in the literature describing new applications of ELPs appear regularly. In addition to the technologies mentioned above, ELP can be used for microarrays (Lee et al. 2009). Megeed et al. 2006) demonstrate the possibility of using ELPs to modulate the affinity of antibodies in response to changes in environmental conditions, which may have applications in the production of biosensors, drug carriers or bioseparation.
